# ARAP1-AS1: a novel long non-coding RNA with a vital regulatory role in human cancer development

**DOI:** 10.1186/s12935-024-03435-w

**Published:** 2024-08-01

**Authors:** Jialing Wang, Hongliang Luo, Lu Yang, Huazhao Yuan

**Affiliations:** 1 Department of General Surgery, Jiujiang Hospital of Traditional Chinese Medicine, Jiujiang, Jiangxi Province 332007 P.R. China; 2 Department of Gastrointestinal Surgery, The Second Affiliated Hospital, Jiangxi Medical College, Nanchang University, Nanchang, Jiangxi 330008 China; 3 Department of Cardiology, The Second Affiliated Hospital, Jiangxi Medical College, Nanchang University, Nanchang, Jiangxi 330008 China

**Keywords:** ARAP1-AS1, Cancer biomarker, Biological functions, Tumor progression

## Abstract

Long non-coding RNAs (lncRNAs) have garnered significant attention in biomedical research due to their pivotal roles in gene expression regulation and their association with various human diseases. Among these lncRNAs, ArfGAP With RhoGAP Domain, Ankyrin Repeat, And PH Domain 1 - Antisense RNA 1 (ARAP1-AS1) has recently emerged as an novel oncogenic player. ARAP1-AS1 is prominently overexpressed in numerous solid tumors and wields influence by modulating gene expression and signaling pathways. This regulatory impact is realized through dual mechanisms, involving both competitive interactions with microRNAs and direct protein binding. ARAP1-AS1 assumes an important role in driving tumorigenesis and malignant tumor progression, affecting biological characteristics such as tumor expansion and metastasis. This paper provides a concise review of the regulatory role of ARAP1-AS1 in malignant tumors and discuss its potential clinical applications as a biomarker and therapeutic target. We also address existing knowledge gaps and suggest avenues for future research. ARAP1-AS1 serves as a prototypical example within the burgeoning field of lncRNA studies, offering insights into the broader landscape of non-coding RNA molecules. This investigation enhances our comprehension of the complex mechanisms that govern the progression of cancer.

**Supplementary Information:**

The online version contains supplementary material available at 10.1186/s12935-024-03435-w.

## Introduction

Cancer is a multifaceted and diverse malignant disease, ranking among the foremost global causes of mortality [1–4]. Genome sequencing projects have shown that while approximately 98% of the human genome is non-coding, resulting in a substantial abundance of non-coding transcripts [5–7]. With the advancement of sequencing technology, a large number of long non-coding RNAs (lncRNAs) have been discovered and identified [8–10], and research has also demonstrated the significant involvement of lncRNAs in the onset and progression of human ailments [11–16], especially tumors [17–21]. As oncogenic or anticancer genes, lncRNAs regulate neoplastic properties such as proliferation, migration, and metastasis, thereby participating in tumor initiation and progression [22–28].

ARAP1-AS1, officially fully known as the ArfGAP With RhoGAP Domain, Ankyrin Repeat, And PH Domain 1 - Antisense RNA 1, exemplifies an RNA gene that falls under the category of lncRNAs. This gene is located on the 11nd chromosome of the human genome, specifically at q13.4. There is only one splice variant that derived from the ARAP1-AS1 gene with 388 bp (https://www.ensembl.org/Homo_sapiens/Gene/Summary?g=ENSG00000256007;r=11:72685075-72693808;t=ENST00000542022). Furthermore, when queried in the NONCODE database (http://www.noncode.org/) [29], it was observed that ARAP1-AS1 exhibits expression exclusively within specific normal human tissues, including adrenal glands, testicles, breasts, colon, and kidneys. However, the expression levels are notably low or undetectable in other tissues. According to data from the lncLocator database (http://www.csbio.sjtu.edu.cn/bioinf/lncLocator/) [30]　and Lnc2Cancer 3.0 (http://www.bio-bigdata.net/lnc2cancer or http://bio-bigdata.hrbmu.edu.cn/lnc2cancer) database [31], ARAP1-AS1 is mainly located in the cytoplasm.

Recently, ARAP1-AS1 has been reported to exhibit upregulation in diverse tumor tissues, including lung cancer [32–34], lymphoma [35], thyroid cancer [36], bladder cancer [37–39], cervical cancer [40–42], ovarian cancer [43], kidney cancer [44], breast cancer [45], gastric cancer [46], and colorectal cancer [47]. ARAP1-AS1 is also implicated in the initiation and advancement of tumorigenesis. However, the precise molecular mode of action by which ARAP1-AS1 exerts its oncogenic potential remains elusive. This article reviews the current evidence on abnormal expression of ARAP1-AS1 and its clinical significance, and further summarizes the regulatory mechanism of ARAP1-AS1 and its role in human carcinogenesis.

### Elevated expression of ARAP1-AS1 across tumor tissues and cell lines

The Cancer Genome Atlas (TCGA) is an important research initiative focused on comprehensively elucidating genomic alterations in various cancer types [48], including alterations associated with ncRNAs, such as lncRNAs. By collecting diverse cancer samples and employing high-throughput genomics technologies, including microarrays and second-generation sequencing, TCGA helps advance detailed studies in cancer research and lncRNA exploration [49, 50]. This approach enhances our understanding of cancer biology and provides insights for personalized cancer treatment [48].

In 2019, Teng et al. [39] conducted an analysis utilizing data from the TCGA database. Their study revealed a significant upregulation of the novel lncRNA, ARAP1-AS1, in bladder cancer samples. This finding was further substantiated through quantitative reverse transcription polymerase chain reaction (qRT-PCR) analysis performed on 88 pairs of bladder cancer tissues and their corresponding non-tumorous tissues. Notably, this study marked the initial documentation of ARAP1-AS1’s relevance in bladder cancer. Subsequently, in the same year, Xu et al. [38] conducted a comprehensive characterization of differentially expressed RNA profiles in bladder cancer using TCGA data. They also identified ARAP1-AS1 as one of the prominently regulated lncRNAs. These observations underscore its potential involvement in the oncogenic processes associated with bladder cancer.

**Fig. 1:**
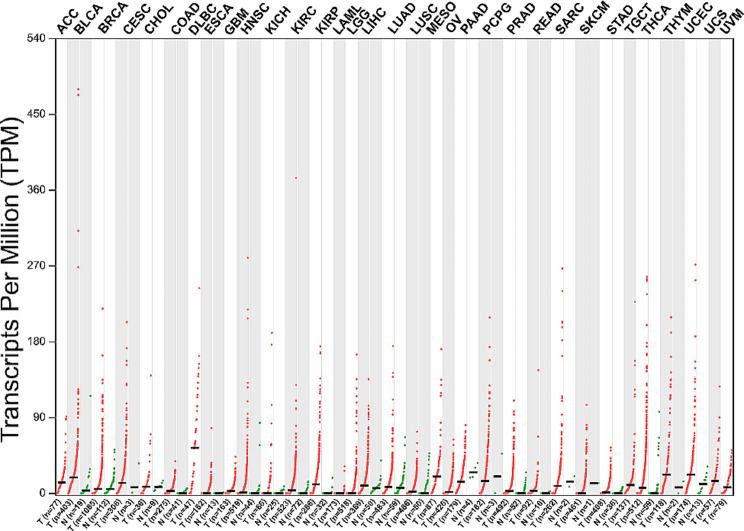
Expression analysis of ARAP1-AS1 in matched TCGA cancer and normal tissues using GEPIA2 tool. The expression of ARAP1-AS is significantly up-regulated in most cancer tissues, while the expression of ARAP1-AS is relatively very low in majority of normal tissues. The abbreviations in this figure can be found in Supplementary Table 1 for reference

Subsequently, multiple research groups have consistently reported a substantial upregulation of ARAP1-AS1 expression in solid tumor specimens originating from a diverse spectrum of cancer types. These encompass colorectal cancer [47], gastric cancer [46], breast cancer [45], cervical cancer [40, 41], lung cancer [32, 34], ovarian cancer [43, 51], renal cancer [44], thyroid cancer [36], and lymphoma [35]. Furthermore, heightened expression of ARAP1-AS1 has been consistently identified in various cancer cell lines pertaining to the aforementioned tumor types. For instance, Ye et al. [47] conducted a study involving 82 pairs of colorectal cancer (CRC) tissues and adjacent non-tumor tissues, revealing frequent overexpression of ARAP1-AS1 in CRC samples. They also observed higher ARAP1-AS1 expression in five CRC cell lines when compared to normal colorectal epithelial cells HCoEpiC. In cervical cancer, Zhang et al. [42] reported a significant upregulation of ARAP1-AS1 in cancer tissues and tumor cell lines. Remarkably, they extended their investigation to peripheral blood samples from both cervical cancer patients and healthy controls. Their findings revealed an eightfold increase in ARAP1-AS1 expression in cervical cancer serum in comparison to serum from healthy controls. In addition, we also comprehensively evaluate the expression of ARAP1-AS1 in pan-cancer using the GEPIA 2 online database tool (http://gepia2.cancer-pku.cn/#index) [52]. As shown in Fig. 1, the expression of ARAP1-AS is significantly up-regulated in most cancer tissues, while the expression of ARAP1-AS is relatively very low in majority of normal tissues. These collective observations underscore the consistent upregulation of ARAP1-AS1 expression in malignant tumors originating from diverse tissues and organs. Consequently, ARAP1-AS1 emerges as a novel oncogenic candidate, with the potential to serve as a tumor biomarker applicable to various cancer types.

### ARAP1-AS1 as a novel tumor-related clinical marker

ARAP1-AS1 emerges as a promising molecular marker with clinical value across various solid tumors. Numerous research groups have explored the relationship between ARAP1-AS1 expression, clinical parameters, and prognosis in cancer, as summarized in Table 1. For instance, in colorectal cancer (CRC) tissues, ARAP1-AS1 expression exhibited significant correlations with TNM stage and lymph node metastasis [47]. Additionally, a preliminary investigation by Jiang et al. [46] unveiled a positive association between ARAP1-AS1 expression and TNM stage and lymphatic metastasis, with higher ARAP1-AS1 levels predicting poorer overall survival (OS) and disease-free survival (DFS) in gastric cancer cases. Furthermore, elevated ARAP1-AS1 levels were indicative of advanced pathological characteristics, including higher FIGO stage and larger tumor size in cervical cancer. Notably, overexpression of ARAP1-AS1 was associated with diminished OS in ovarian cancer, cervical cancer, bladder cancer, and lymphoma. Intriguingly, serum ARAP1-AS1 demonstrated good diagnostic potential for cervical cancer patients, with an area under the curve (AUC) value of 0.8953. ARAP1-AS1 harbors significant promise for tumor diagnostic detection and prognostic assessment; however, further clinical investigations remain imperative to solidify its applications in the clinical setting.

**Table 1:** The expression of ARAP1-AS1 in tumor samples and tumor cell lines, its clinical significance in relation to clinical features, and its prognostic and diagnostic implications

Tumor type	Expression in tumor tissues	Clinical features	Prognosis	Diagnosis	Expression in cancer cell lines	Reference
Lung adenocarcinoma	Upregulated	/	/	/	Upregulated	[32]
	Upregulated	/	/	/	Upregulated	[33]
Lung cancer	Upregulated	/	/	/	Upregulated	[34]
Lymphoma	Upregulated	/	OS (*P* = 0.0880)	/	Upregulated	[35]
Thyroid cancer	Upregulated	/	/	/	Upregulated	[36]
Bladder cancer	Upregulated	/	/	/	Upregulated	[37]
	Upregulated	/	OS (*P* = 0.029)	/	Upregulated	[39]
Cervical cancer	Upregulated	FIGO stage, tumor size	/	/	Upregulated	[40]
	Upregulated	/	/	/	Upregulated	[41]
	Upregulated	Tumor size, FIGO stage, lymph node metastasis	OS (*P* = 0.0098)	AUC: 0.8953	Upregulated	[42]
Ovarian cancer	Upregulated	/	OS (*P* = 0.017)	/	Upregulated	[43]
Clear cell renal cell carcinoma	Upregulated	/	/	/	Upregulated	[44]
Breast cancer	Upregulated	/	/	/	Upregulated	[45]
Gastric cancer	Upregulated	TNM stage, lymphatic metastasis	OS (*P* = 0.0078); DFS (*P* = 0.0020)	/	/	[46]
Colorectal cancer	Upregulated	TNM, lymph node metastasis	/	/	Upregulated	[47]

FIGO stage: International Federation of Gynecology and Obstetrics stage; TNM stage: Tumor, Node, Metastasis stage; OS: Overall Survival; DFS: Disease-Free Survival; AUC: Area Under the Curve; “ / ”: Indicates missing or not applicable data

### Functional role and regulatory mechanisms of ARAP1-AS1 in tumor development

**Fig. 2:**
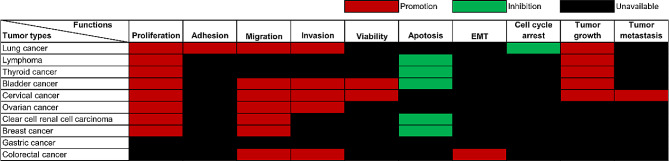
The functions of oncogenic ARAP1-AS1 in different cancers. This figure offers a detailed overview of the roles of ARAP1-AS1 in a variety of tumor types. It underscores the wide-ranging functions of ARAP1-AS1 in diverse cancers, including lung cancer, lymphoma, thyroid cancer, bladder cancer, among others. These functions encompass the regulation of crucial cellular processes such as proliferation, adhesion, invasion, tumor metastasis, and more

ARAP1-AS1 consistently demonstrates upregulation in a multitude of tumor tissues and cell lines, collectively emphasizing its potential oncogenic role across various tumor types. Studies have explored the biological role of ARAP1-AS1 in different tumors in vivo and/or in vitro experimental approaches, as documented in Table 2. ARAP1-AS1 is involved in a series of tumor-related biological processes (Fig. 2). Specifically, ARAP1-AS1 acts as a potent promoter of epithelial-mesenchymal transition (EMT), an enhancer of cellular proliferation and viability, a facilitator of cell migration and invasion, a catalyst for tumor growth, and a potent contributor to metastasis. Conversely, ARAP1-AS1 exerts inhibitory effects on apoptosis and hinders cell cycle arrest in tumor cells. These findings collectively highlight the multifaceted role of ARAP1-AS1 as a key oncogenic regulator with significant impact on tumor initiation and progression.

**Table 2:** Function and regulatory mechanisms of ARAP1-AS1 in tumors using in vitro and/or in vivo experiments

Tumor type	Experiments	Regulatory mechanism	ARAP1-AS1 function	Effects in vitro	Effects in vivo	Reference
Lung adenocarcinoma	In vitro and in vivo	ARAP1-AS1/miR-8068/CEACAM5	ceRNA	proliferation, adhesion, migration	Tumor growth	[32]
	In vitro	ARAP1-AS1/ EZH2/ARAP1	Epigenetic regulation	proliferation, migration, invasion	/	[33]
Lung cancer	In vitro and in vivo	/	/	Proliferation, cell cycle arrest	Tumor growth	[34]
Lymphoma	In vitro and in vivo	ARAP1-AS1/miR-6867-5p	sponge	cell proliferation, apoptosis	Tumor growth	[35]
Thyroid cancer	In vitro and in vivo	ARAP1-AS1/miR-516b-5p/PDE5A	ceRNA	cell proliferation, apoptosis	Tumor growth	[36]
Bladder Cancer	In vitro and in vivo	ARAP1-AS1/miR-3918 /KIF20A	ceRNA	cell proliferation, apoptosis, viability	Tumor growth	[37]
	In vitro and in vivo	ARAP1-AS1/miR-4735-3p/NOTCH2	ceRNA	cell viability, proliferation, migration, invasion	Tumor growth	[39]
Cervical cancer	In vitro and in vivo	ARAP1-AS1/miR-149-3p /POU2F2	ceRNA	Proliferation, migration, invasion	Tumor growth and metastasis	[40]
	In vitro	ARAP1-AS1/ EZH2/DUSP5	Epigenetic regulation	cell viability, proliferation, migration	/	[41]
	In vitro and in vivo	ARAP1-AS1/ PSF/PTB dimer/c-Myc	Protein decoy	cell viability, proliferation, invasion	Tumor growth and metastasis	[42]
Ovarian cancer	In vitro	ARAP1-AS1/miR-4735-3p/PLAGL2	ceRNA	proliferation, migration, invasion	/	[43]
Clear cell renal cell carcinoma	In vitro	ARAP1-AS1/miR-361-3p /PGF	ceRNA	proliferation and migration, apoptosis	/	[44]
Breast cancer	In vitro	ARAP1-AS1/miR-2110/HDAC2/ PLIN1	ceRNA	proliferation, migration, apoptosis	/	[45]
Colorectal Cancer	In vitro	YY1/ARAP1-AS1/Wnt/β-catenin pathway	/	migration, invasion, EMT	/	[47]

ceRNA: Competing Endogenous RNA; EMT: Epithelial-Mesenchymal Transition; “ / ”: Indicates missing or not applicable data

**Fig. 3:**
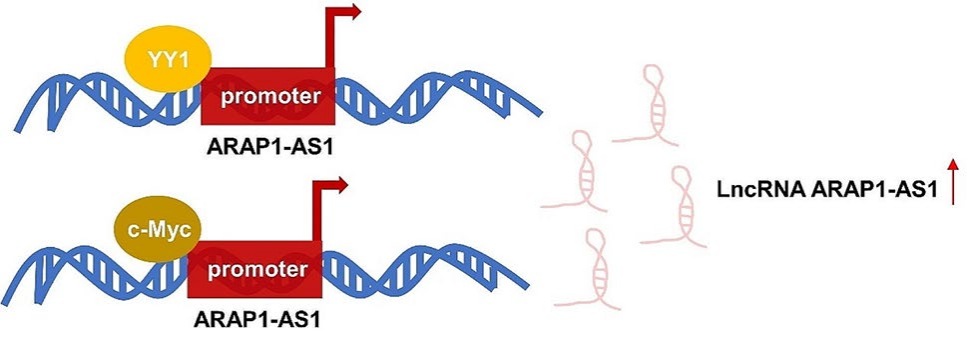
Mechanism responsible for the upregulated expression of ARAP1-AS1 in tumors. Certain transcription factors, including YY1 and c-Myc, attach themselves to the ARAP1-AS1 promoter, thereby initiating the transcription of ARAP1-AS1

Mounting evidence suggests that lncRNAs can be triggered into action by the transcription factors situated upstream of them. Notably, the promoter region of ARAP1-AS1 contains binding sites for a number of transcription factors, such as c-Myc and YY1 (as depicted in Fig. 3). To delve deeper, the study by Ye et al. [47] revealed that YY1 has the capability to enhance ARAP1-AS1 expression by promoting its transcription. Furthermore, it was uncovered that the Wnt/β-catenin signaling pathway operates downstream of ARAP1-AS1. This YY1/ARAP1-AS1 axis was found to drive colorectal cancer cell migration by activating the Wnt/b-catenin signaling pathway. Another investigation by Zhang et al. [42] documented that c-Myc can activate ARAP1-AS1 at the transcriptional level by directly binding to the E-box motif located within the ARAP1-AS1 promoter. This discovery led to the identification of a positive feedback loop between ARAP1-AS1 and c-Myc, amplifying the carcinogenic impact of ARAP1-AS1.

The regulatory mechanisms associated with ARAP1-AS1 in tumor development are complex and multifaceted. In addition to its involvement in ceRNA networks and protein binding (acting as a modular scaffold or protein decoy), ARAP1-AS1 can also regulate gene expression through multiple mechanisms, affecting the behavior of cancer cells. The specific details of these mechanisms are complex and require further exploration. For instance, in the context of breast cancer [45], ARAP1-AS1 was observed to enhance the expression of HDAC2 by binding to miR-2110. Subsequently, HDAC2 led to the transcriptional silencing of PLIN1 through deacetylation of the PLIN1 promoter. Moreover, it was confirmed that ARAP1-AS1 exerts a negative regulatory effect on PLIN1 expression. Thus, ARAP1-AS1 exacerbates the development of breast cancer by inducing the transcriptional suppression of PLIN1 through the deacetylation activity of HDAC2 on the PLIN1 promoter. Furthermore, Min et al. [34] observed that the increased expression of ARAP1-AS1 led to G0/G1 cell cycle arrest in lung cancer cells by suppressing cyclin D1 expression. Nevertheless, the precise mechanism through which ARAP1-AS1 modulates cyclin D1 expression, whether via miRNA or other genetic proteins, warrants additional investigation. All these findings underscore the multifaceted regulatory roles of ARAP1-AS1 in influencing gene expression, consequently, contributing regulate various biological processes in different cancer types.

### LncRNA ARAP1-AS1’s participation in the competing endogenous RNA network

ARAP1-AS1 exerts control over the expression of downstream genes by engaging in competitive interactions with miRNAs, a fundamental component of the competing endogenous RNA (ceRNA) regulatory mechanism, as previously explained in earlier studies [53–55]. The ceRNA hypothesis proposes that extended RNA molecules, including both lncRNAs and mRNAs, which share common miRNA binding sites, participate in dynamic competitions for miRNA binding, thereby mutually influencing each other’s expression [56–58].

**Fig. 4:**
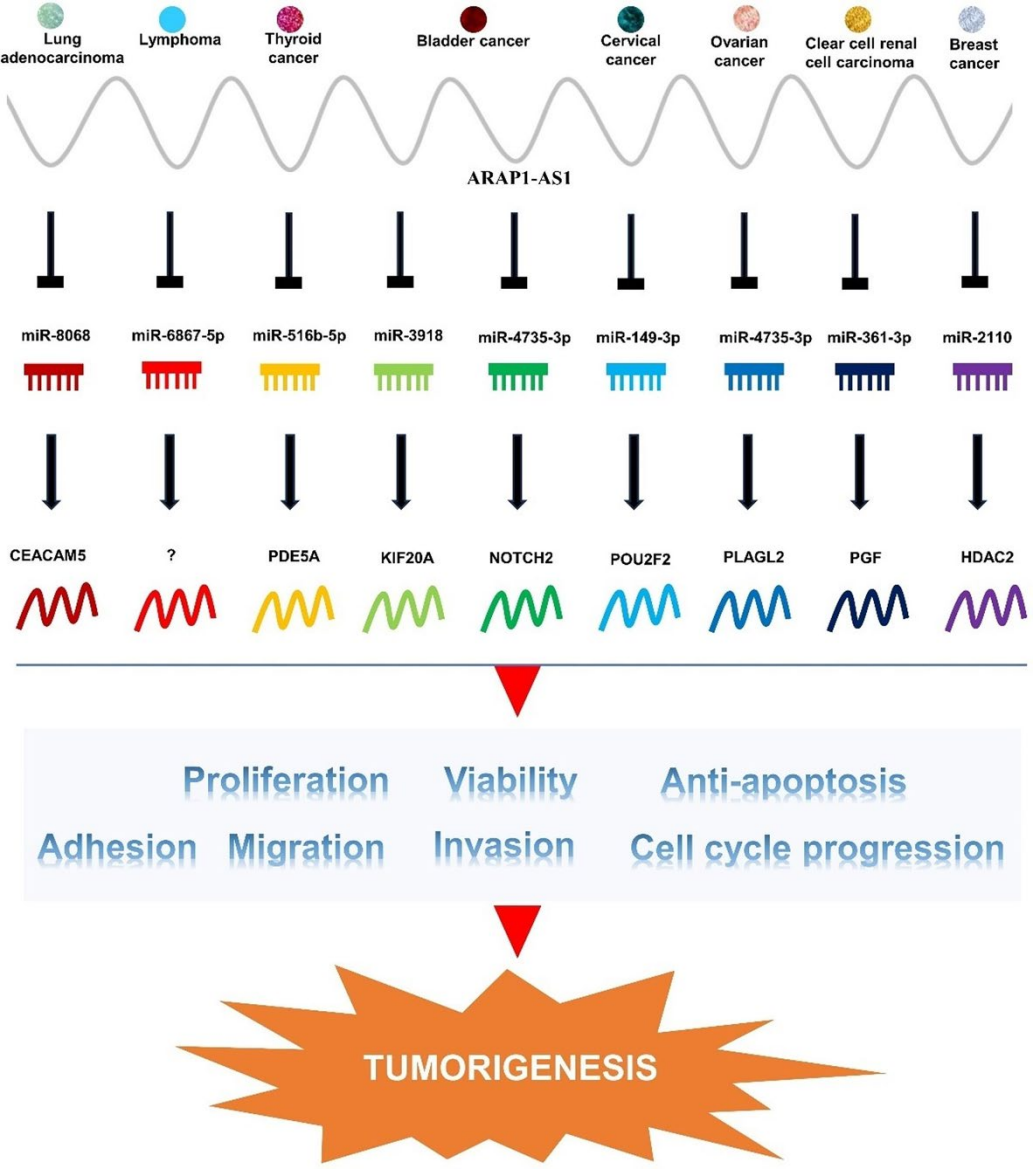
ARAP1-AS1 engages in competitive binding with miRNAs, thereby orchestrating the modulation of various mRNA expressions that drive tumor progression. ARAP1-AS1 competes for miRNAs such as miR-8068, miR-516b-5p, miR-3918, among others, resulting in the upregulation of mRNAs such as CEACAM5, PDE5A, KIF20A, and others. This molecular interplay promotes malignant characteristics like proliferation, invasion, and migration, ultimately fueling the initiation of tumorigenesis

ARAP1-AS1, as an exemplar of lncRNAs, harbors numerous potential miRNA binding sites. This attribute endows ARAP1-AS1 with the capacity to regulate the expression of diverse mRNAs by competitively vying for multiple miRNAs. In turn, this regulation cascades downstream, impacting gene expression and culminating in the modulation of malignant phenotypes in tumors. Across different tumor types, ARAP1-AS1 exhibits the versatility to competitively bind various miRNAs, including miR-8068, miR-516b-5p, miR-3918, miR-4735-3p, miR-149-3p, miR-361-3p, miR-2110, and more (Fig. 4). Consequently, this competition leads to the upregulation of corresponding mRNA expression, involving genes such as CEACAM5, PDE5A, KIF20A, NOTCH2, POU2F2, PLAGL2, PGF, and HDAC2 (Fig. 4). The ensuing synthesis of these proteins contributes to the orchestration of malignant phenotypes, encompassing tumor proliferation, invasion, migration, thereby fueling the initiation and progression of tumors. It is noteworthy that ARAP1-AS1’s potential to bind additional miRNAs for the modulation of tumor development remains an intriguing avenue that warrants further exploration.

### LncRNA ARAP1-AS1 modulates downstream gene expression via direct protein binding

**Fig. 5:**
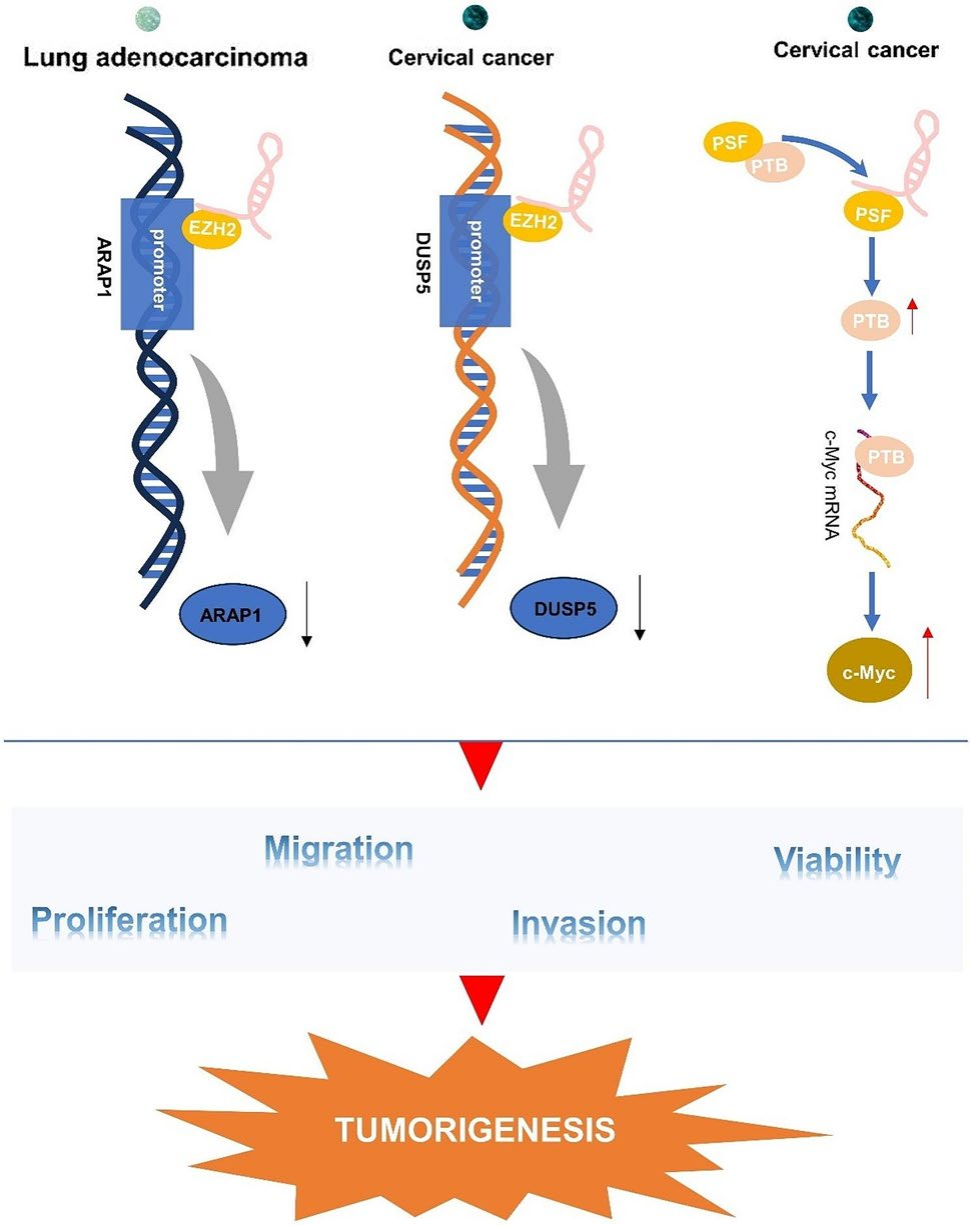
ARAP1-AS1 promotes tumor development by binding to proteins. ARAP1-AS1 epigenetically silences ARAP1 or DUSP5 by recruiting EZH2 to promote tumorigenesis of lung adenocarcinoma and cervical cancer, respectively, and ARAP1-AS1 can directly interact with PSF to release PTB, thereby accelerating c-Myc translation, and promoting occurrence of cervical cancer

Apart from competing with miRNAs and regulating mRNA stability, lncRNA ARAP1-AS1 could form complexes with specific proteins, acting at the epigenetic and transcriptional levels to control gene expression (Fig. 5). In a study by Liu et al. [33], it was uncovered that ARAP1-AS1 silences ARAP1 expression by recruiting EZH2 to its promoter region, leading to increased enrichment of H3K27me on the promoter. Consequently, the suppression of ARAP1 expression promotes cell proliferation, migration, and invasion in lung adenocarcinoma. In the context of cervical cancer, ARAP1-AS1 recruits EZH2 to the DUSP5 promoter, resulting in epigenetic silencing of DUSP5. The suppression of DUSP5 through EZH2-mediated methylation further enhances cervical cancer cell proliferation and migration [41]. Additionally, Zhang et al. [42]reported that ARAP1-AS1 directly interacts with PSF to release PTB, which accelerates the translation of c-Myc, ultimately facilitating the development of progression of cervical cancer.

The ARAP1-AS1 RNA function is intricately tied to its secondary structure. Through an examination of the secondary structure of ARAP1-AS1, we have the potential to unveil additional proteins associated with its role in tumor regulation. Additionally, it’s worth noting that ARAP1-AS1 has been documented to reside both in the cytoplasm and the nucleus. Conducting subcellular localization analyses of ARAP1-AS1 in various tissues provides yet another avenue for delving into its mechanisms of tumor development regulation.

### Summary and future perspectives

The realm of cancer research has witnessed significant advancements with the discovery and study of lncRNAs, which play crucial roles in the regulation of gene expression and the development of various malignancies [59–61]. Among these, ARAP1-AS1, a recently identified lncRNA, has garnered attention for its involvement in tumor progression and potential as a biomarker for cancer diagnosis and prognosis. This review summarized the current understanding of ARAP1-AS1’s mechanisms of action, the limitations of existing studies, and propose directions for future research.

ARAP1-AS1 plays a multifaceted role in the regulation of tumorigenesis through its complex interactions at the molecular level. It primarily acts as a miRNA sponge, effectively sequestering miRNAs and preventing them from inhibiting their target oncogenes. Additionally, ARAP1-AS1 serves as a decoy for proteins, modulating the activity of RNA-binding proteins crucial for post-transcriptional regulation. These strategic interactions demonstrate that ARAP1-AS1 regulates gene expression at the epigenetic and transcriptional level, contributing further to the modulation of related signaling pathways. ARAP1-AS1 has been established as a carcinogenic lncRNA in numerous tumor types. Elevated ARAP1-AS1 expression significantly promotes malignant traits like cell proliferation, invasion, and migration in various tumors. Conversely, downregulating ARAP1-AS1 has opposing effects. These findings underscore the potential of ARAP1-AS1 as a vital anti-tumor target, and inhibiting its expression may enhance therapeutic efficacy in cancer patients. Increasingly, studies have reported the use of small molecule drugs derived from natural products to inhibit the expression of oncogenic lncRNAs, such as cucurbitacin B [62] and oridonin [63] targeting AFAP1-AS1. Thus, the exploration of small molecule compounds, particularly those from natural sources, that can inhibit ARAP1-AS1 expression holds promise as a meaningful avenue of research.

Despite these insights, there are significant gaps in our understanding of ARAP1-AS1. Many studies to date have relied heavily on in vitro assays and limited clinical investigations, highlighting the necessity for comprehensive in vivo studies and more extensive, diverse clinical trials. These studies are crucial for validating the molecular interactions and functions related to ARAP1-AS1 in a broader range of cancers, not only on human solid tumors but also in hematological malignancies. Additionally, the exploration of ARAP1-AS1’s role in larger ceRNA networks and additional tumor-related signaling pathways in different cancers remains an underexplored area with considerable potential for future research. Nevertheless, it is imperative to delve into the intricate upstream and downstream molecular mechanisms of ARAP1-AS1 to elucidate its precise role in tumor regulation. Moreover, the retrospective nature of some studies and the use of small sample sizes for evaluating ARAP1-AS1’s diagnostic and prognostic value are significant limitations. These issues underscore the need for larger, prospective studies to establish the clinical utility of ARAP1-AS1 more firmly.

To enhance this review, we leveraged multiple online lncRNA databases and high-throughput datasets to uncover the clinical relevance and potential pathway engagement of ARAP1-AS1 in the tumorigenesis and disease progression.

**Fig. 6:**
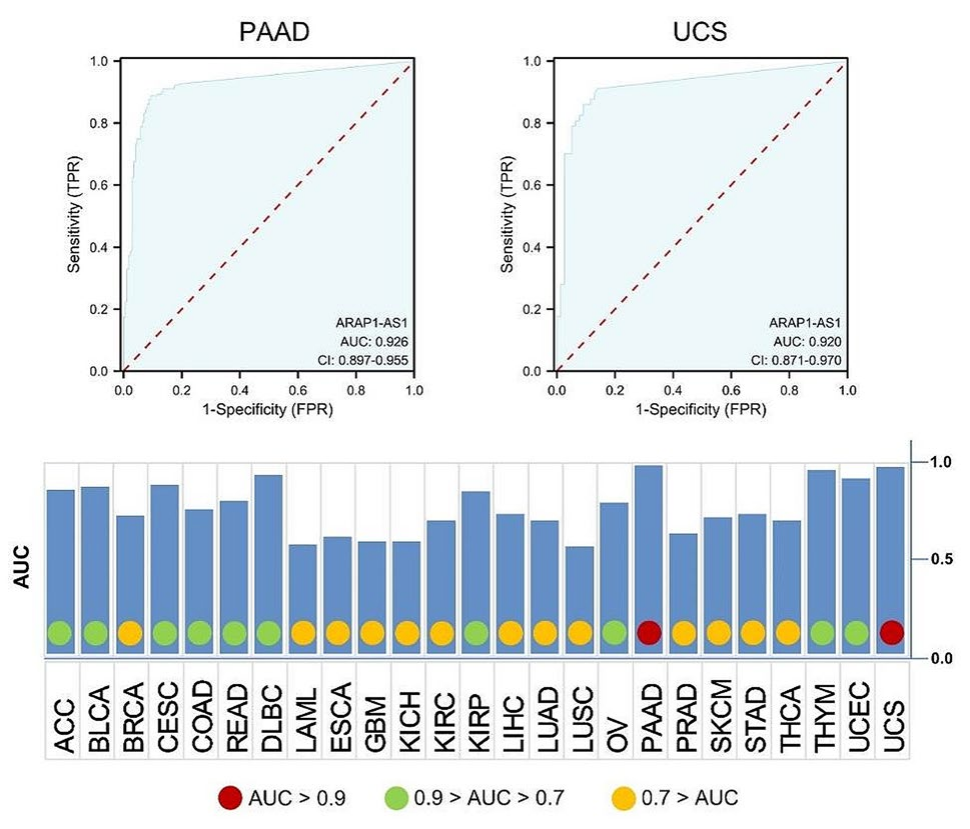
Diagnostic receiver operating characteristic curves for ARAP1-AS1 expression in distinguishing tumors from normal tissues across a broad spectrum of cancers. Notably, ARAP1-AS1 expression demonstrated significant diagnostic potential in PAAD and UCS. The abbreviations in this figure can be found in Supplementary Table 1 for reference

**Fig. 7:**
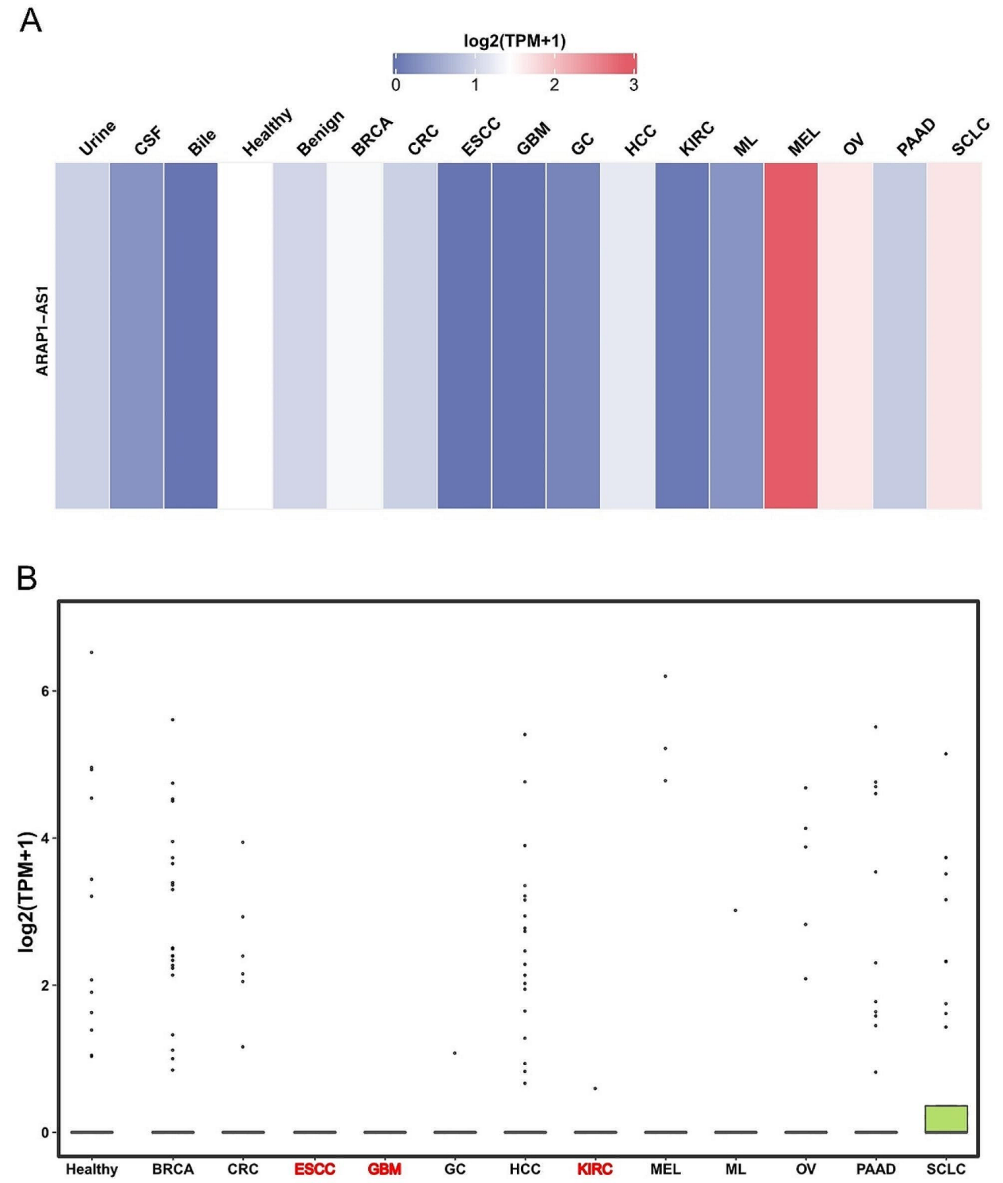
(**A**) Heat map representation of ARAP1-AS1 expression levels within extracellular vesicles, as determined by RNA sequencing analyses across a variety of human bodily fluids. (**B**) Comparative analysis of ARAP1-AS1 expression in extracellular vesicles from the blood of individuals with various types of cancer relative to healthy donors. Cancer types demonstrating significantly different ARAP1-AS1 expression levels when compared to those of healthy donors are marked in red. The abbreviations in this figure can be found in Supplementary Table 1 for reference

While the diagnostic utility of ARAP1-AS1 expression has been previously documented in cervical cancer [42], we extend this investigation to a broader array of tumor types. We analyzed data from the UCSC XENA platform (https://xenabrowser.net/datapages/), uncovering that ARAP1-AS1 expression possesses the potential to serve as a robust diagnostic biomarker across multiple cancers (Fig. 6). Notably, in pancreatic adenocarcinoma (PAAD) and uterine carcinosarcoma (UCS), ARAP1-AS1 demonstrated an AUC exceeding 0.9, highlighting its significant diagnostic promise　(Fig. 6). Our focus then shifted towards evaluating ARAP1-AS1’s potential as a minimally invasive diagnostic instrument. Given the emerging recognition of exosomal lncRNAs as viable tumor diagnostic markers [64–66], and considering that ARAP1-AS1’s expression in blood samples has been reported only in cervical cancer [42], we utilized exoRBase 2.0 (http://www.exorbase.org/) [67]—a repository for extracellular vesicles long RNAs derived from RNA-seq data—to assess the expression of lncRNA across various human body fluids, including blood, urine, cerebrospinal fluid (CSF), and bile. Our analysis revealed that ARAP1-AS1 levels vary under different disease conditions, with the highest levels observed in the blood of melanoma (MEL) patients (Fig. 7A). A subsequent comparative analysis of ARAP1-AS1 expression in the blood of cancer patients versus healthy donors revealed significant differences in esophageal squamous cell carcinoma (ESCC), glioblastoma multiforme (GBM), and kidney renal clear cell carcinoma (KIRC) (Fig. 7B). This suggests ARAP1-AS1’s potential as a circulating diagnostic lncRNA in these cancers. However, confirmation of ARAP1-AS1 expression and its diagnostic efficacy is needed in larger clinical cohorts across various types of tumors. These observations underscore ARAP1-AS1’s potential as a promising diagnostic marker in select tumors and pave the way for its exploration as a novel circulating biomarker in future research.

**Fig. 8:**
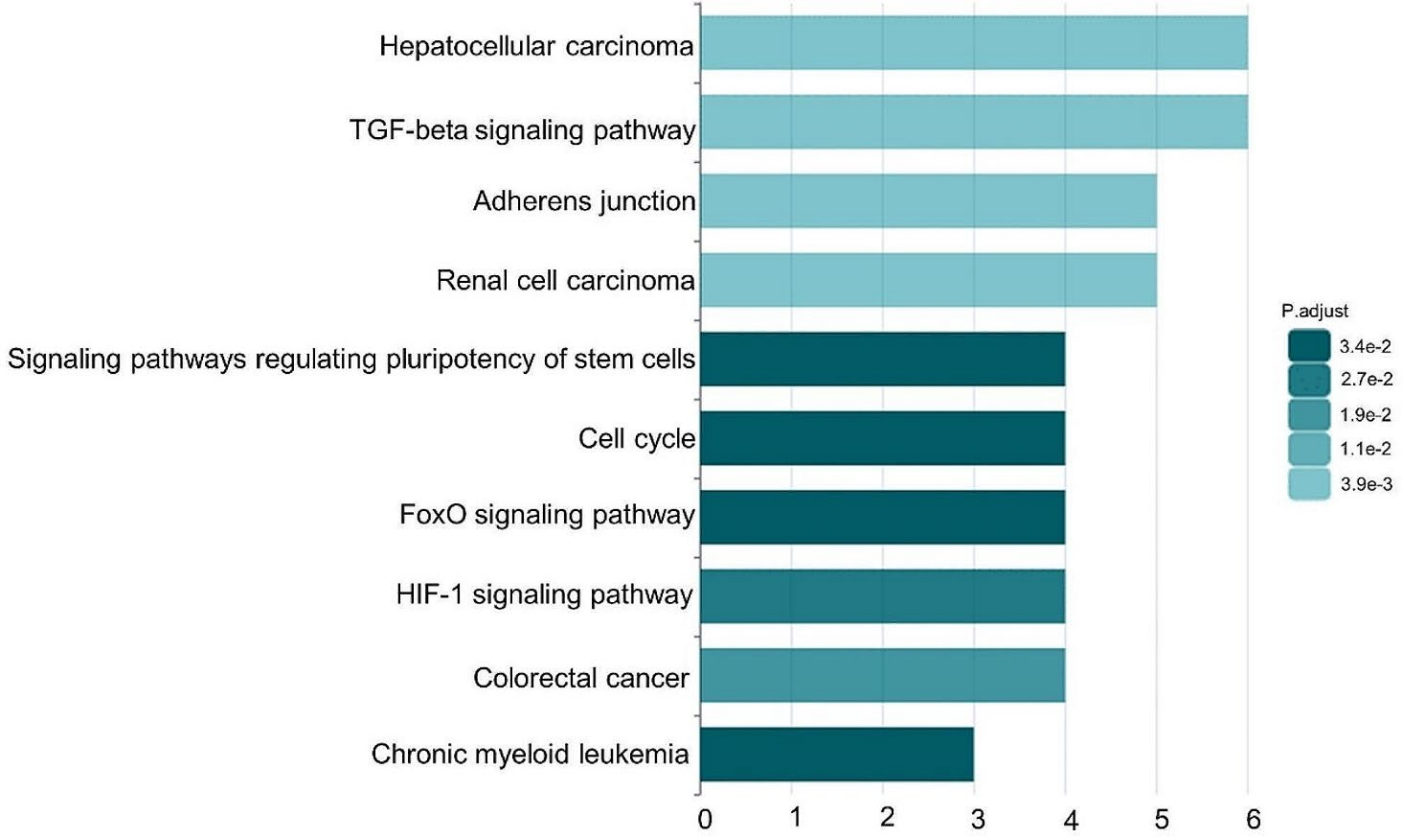
The top ten significantly enriched signaling pathways targeted by ARAP1-AS1

Moreover, we undertook an analysis using NcPath [68], an online platform for the visualization and enrichment analysis of human non-coding RNA and KEGG signaling pathways, to identify the top 10 enriched signaling pathways involving ARAP1-AS1 (Fig. 8). The results illuminated ARAP1-AS1’s association with several cancer-related pathways, notably the TGF-beta signaling pathway, FoxO signaling pathway, and HIF-1 signaling pathway. These pathways are intrinsically linked to the initiation and progression of various tumors [69–76], highlighting the intricate role ARAP1-AS1 in cancer biology and emphasizing its significance in the study of tumorigenesis and cancer progression.

## Conclusion

In summary, ARAP1-AS1, identified as a tumor-promoting lncRNA, exhibits elevated expression levels across various solid tumors and has gained significant attention in non-coding RNA research. It plays a pivotal role in initiating and advancing malignant tumors and holds potential as a molecular marker and therapeutic target for multiple cancer types. Nonetheless, the investigation of ARAP1-AS1 is still in its infancy. Thorough and comprehensive studies into its functions and biological significance are imperative to deepen our understanding of tumor pathogenesis. This expanded knowledge base will prove invaluable in advancing the diagnosis and treatment of cancer.

### Electronic supplementary material

Below is the link to the electronic supplementary material.

Supplementary Material 1

## Data Availability

Not applicable.
