# Correction: ARAP1-AS1: A novel long non-coding RNA with a vital regulatory role in human cancer development

**DOI:** 10.1186/s12935-024-03475-2

**Published:** 2024-08-16

**Authors:** Jialing Wang, Hongliang Luo, Lu Yang, Huazhao Yuan

**Affiliations:** 1 Department of General Surgery, Jiujiang Hospital of Traditional Chinese Medicine, Jiujiang, 332007 Jiangxi Province P.R. China; 2 Department of Gastrointestinal Surgery, The Second Affiliated Hospital, Jiangxi Medical College, Nanchang University, Nanchang, 330008 Jiangxi China; 3 Department of Cardiology, The Second Affiliated Hospital, Jiangxi Medical College, Nanchang University, Nanchang, 330008 Jiangxi China

**Correction to: Cancer Cell International (2024) 24:270**

10.1186/s12935-024-03435-w

In this article [1], Jialing Wang and Hongliang Luo should have been denoted as equally contributing authors.
