# Supplementary material for: ARAP1-AS1: a novel long non-coding RNA with a vital regulatory role in human cancer development
Source: Cancer Cell Int. 2024 Aug 1;24:270. doi: 10.1186/s12935-024-03435-w (PMC11295494; doi:10.1186/s12935-024-03435-w)
Supplement: Supplementary file 1 — Supplementary Material 1 [file 12935_2024_3435_MOESM1_ESM.docx]

**Supplementary Table 1. Abbreviations in Figure 1, 6 and 7.**

| **Abbreviation** | **Full name** |
| --- | --- |
| **ACC** | Adrenocortical carcinoma |
| **BLCA** | Bladder Urothelial Carcinoma |
| **BRCA** | Breast invasive carcinoma |
| **CESC** | Cervical squamous cell carcinoma and endocervical adenocarcinoma |
| **CHOL** | Cholangio carcinoma |
| **COAD** | Colon adenocarcinoma |
| **CSF** | Cerebrospinal fluid |
| **READ** | Rectum adenocarcinoma |
| **DLBC** | Lymphoid Neoplasm Diffuse Large B-cell Lymphoma |
| **ESCC** | Esophageal squamous cell carcinoma |
| **ESCA** | Esophageal carcinoma |
| **GBM** | Glioblastoma multiforme |
| **GC** | Gastric cancer |
| **HCC** | Hepatocellular carcinoma |
| **HNSC** | Head and Neck squamous cell carcinoma |
| **KICH** | Kidney Chromophobe |
| **KIRC** | Kidney renal clear cell carcinoma |
| **KIRP** | Kidney renal papillary cell carcinoma |
| **LAML** | Acute Myeloid Leukemia |
| **LGG** | Brain Lower Grade Glioma |
| **LIHC** | Liver hepatocellular carcinoma |
| **LUAD** | Lung adenocarcinoma |
| **LUSC** | Lung squamous cell carcinoma |
| **MESO** | Mesothelioma |
| **ML** | Malignant lymphoma |
| **MEL** | Melanoma |
| **OV** | Ovarian serous cystadenocarcinoma |
| **PAAD** | Pancreatic adenocarcinoma |
| **PCPG** | Pheochromocytoma and Paraganglioma |
| **PRAD** | Prostate adenocarcinoma |
| **READ** | Rectum adenocarcinoma |
| **SARC** | Sarcoma |
| **SKCM** | Skin Cutaneous Melanoma |
| **STAD** | Stomach adenocarcinoma |
| **SCLC** | Small cell lung cancer |
| **TGCT** | Testicular Germ Cell Tumors |
| **THCA** | Thyroid carcinoma |
| **THYM** | Thymoma |
| **UCEC** | Uterine Corpus Endometrial Carcinoma |
| **UCS** | Uterine Carcinosarcoma |
| **UVM** | Uveal Melanoma |
